# Claims of sex-dependent effects in the behavioral and brain sciences are infrequently supported by valid statistical comparisons

**DOI:** 10.1073/pnas.2608703123

**Published:** 2026-08-04

**Authors:** Madeline T. Olivier, Andrew W. Brown, Simon Chung, Colby J. Vorland, Donna L. Maney

**Affiliations:** ^a^ Department of Psychology, Emory University, Atlanta, GA 30322; ^b^ Department of Biostatistics, University of Arkansas for Medical Sciences, Little Rock, AR 72205; ^c^ Arkansas Children’s Research Institute, Little Rock, AR 72202; ^d^ Department of Epidemiology and Biostatistics, Indiana University School of Public Health-Bloomington, Bloomington, IN 47403

**Keywords:** sex-specific, gender-specific, gender-dependent, sex as a biological variable

## Abstract

Interest in precision medicine has accelerated efforts to identify sex-dependent responses to treatments and interventions. Those efforts have, both historically and today, been concentrated in fields related to the brain and behavior. Here, we show that even though articles claiming sex-dependent effects are far more prevalent in the neurosciences than in other fields, those claims are infrequently supported by appropriate statistical evidence. Of 200 recent articles in the behavioral and brain sciences with a claim of a sex- or gender-dependent effect in the title, only 24% supported the claim with appropriate evidence—that is, they compared the effect statistically across sex and reported results consistent with the claim. In 9%, an appropriate test was described but the results were incomplete or missing; another 9% contained results that directly contradicted the claim. In most articles (58%), the effect was not compared statistically across sex. Across six brain-related research areas (behavioral sciences, clinical neurology, neurosciences, psychiatry, psychology, and substance abuse), neurosciences had the lowest rate of appropriate evidence (18%) and psychology the highest (39%). Studies on nonhuman animals supported the claim significantly less frequently (15%) than studies on human participants (34%; *P* = 0.002). The use of appropriate analytical approaches was unrelated to journal rank or the citation impact of the article. We conclude that claims of sex- or gender-dependent effects in the behavioral and brain sciences are often not supported by appropriate evidence, revealing a widespread inferential gap that may undermine translation of sex-dependent findings into evidence-based treatments.

## Significance

Sex-inclusive research policies and interest in precision medicine have increased efforts to identify sex-dependent responses to treatments and interventions. Here, we show that claims of sex-dependent effects are disproportionately featured in article titles in the neurosciences, compared with other fields, while at the same time, most such claims lack appropriate statistical support. Among 200 recent studies in behavioral and brain sciences claiming a sex- or gender-dependent effect in the title, fewer than one quarter presented statistical evidence consistent with that claim. A majority did not compare responses across sex at all. These findings reveal a gap between the analytical standards required to demonstrate sex-dependent effects and current research practices, raising concerns about the evidentiary basis for sex-specific treatment recommendations.

Advances in public health rest on rigorous research conducted on diverse populations. In recent decades, sex has been singled out as a critical dimension of this diversity, prompting policies and guidelines designed to increase the inclusion of both females and males in biomedical research (1–4). An important rationale for such policies is that the routine exclusion of an entire sex or gender from research design, analysis, and reporting is difficult to reconcile with standards of scientific rigor or broad generalizability (2, 5, 6). Since the implementation of policies such as “Sex as a Biological Variable” (SABV) by the NIH in the U.S. and “Sex and Gender Based Analysis” (SGBA) by the Canadian Institutes of Health Research (CIHR), the proportion of studies that include females and males has increased (7–11), suggesting that the policies are having a positive effect on sex inclusion.

Beyond concerns about generalizability, sex-inclusive research policies are grounded in the view that men and women differ systematically in patterns of disease, treatment response, and adverse drug effects, and that those differences remain largely undocumented (2, 5, 6, 12). Toward the goal of revealing differences, the policies call for researchers to disaggregate and analyze data by sex (3). Comparing the sexes formally is not required; however, in compliant research, comparative language (e.g., females responded more than males; the sexes were similar) is used more than 80% of the time and conclusions of difference are often highlighted in titles or abstracts (13, 14). Recent literature analyses suggest a lapse in analytical rigor, particularly in studies designed to show a sex-dependent response to a treatment or intervention (9, 13–15).

In this study, we conducted a targeted analysis of analytical approaches to sex-dependent effects. Unlike other recent literature analyses of sex as a variable, this investigation focused exclusively on articles in which sex-dependent effects were reported and emphasized: in all articles, the effect was featured in the title, indicating that it was considered a principal finding. We further focused on research areas in which sex differences have historically been emphasized and remain culturally salient—namely, the behavioral and brain sciences (16–19). In these domains, where claims of sex differences are highly visible, rigorous statistical support is especially important. These articles therefore represent a corpus in which we should expect explicit and convincing statistical evidence supporting the title claim.

## Results

### Terminology and Scope.

#### Behavioral and brain sciences.

Our goal was to sample broadly from literature relevant to behavior and the brain. Our corpus consisted of articles tagged in Web of Science with research categories in the following general areas: behavioral sciences, clinical neurology, neurosciences, psychiatry, psychology, and substance abuse. We refer to these research areas as our “focal” areas. See Dataset S1 for a complete list of all research area tags.

#### Sex and gender.

The terms “sex” and “gender” emerged from different scholarly traditions and are not equivalent (20). Their operationalization can vary according to research question and other contextual factors (21). Here, we use the terms to refer to the binary categories to which animals or participants were assigned in the articles in our sample. The two terms are generally conflated in the literature (22–24) and meta-research typically includes both as search terms (e.g., refs. 15 and 25). Because no study in our sample included both sex and gender as variables in analyses, treating them as equivalent in this case does not result in loss of generality. In this article, we use the term sex to refer to sex or gender, “males” to refer to males, men, or boys, and “females” to refer to females, women, or girls.

#### Effect.

We adopt the term “effect” to refer to the influence or association of a nonsex factor that has been tested in males and females. “Effect” is commonly used in this context, regardless of whether an investigation is causal or associative in nature. For example, a “sex-dependent effect” could refer to the effect of an experimental factor that depends on whether mice are female or male; it could also refer to an association between two variables, such as a risk factor and a health outcome, that differs between men and women.

### Article Characteristics.

**Fig. 1.**
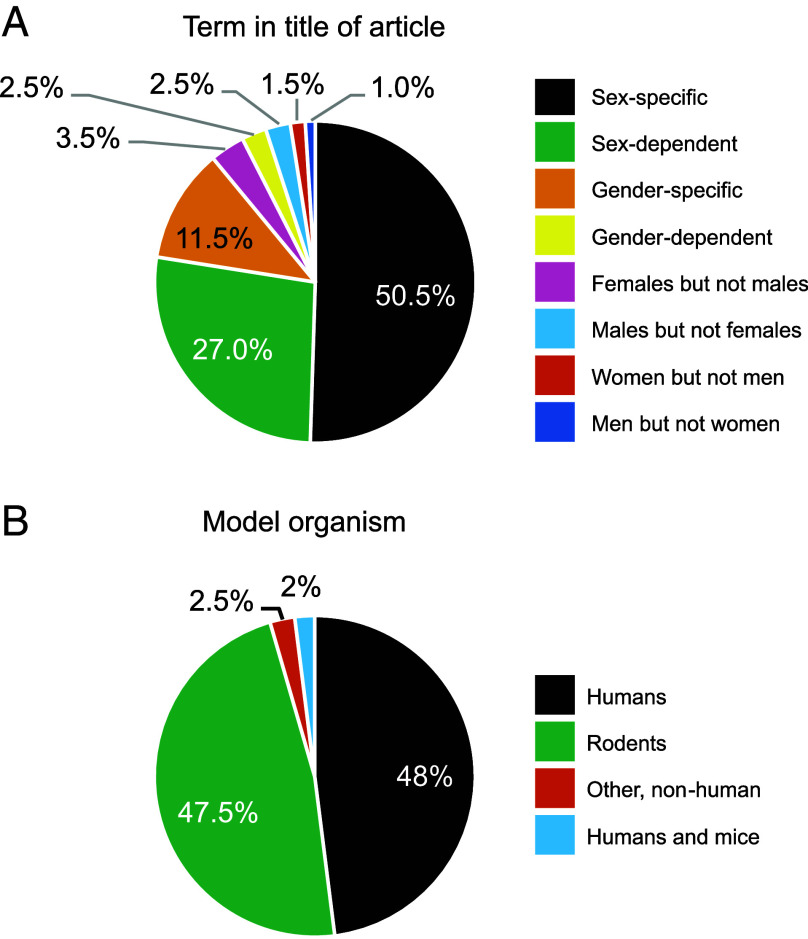
Title terms and model organisms in a sample of 200 articles in the behavioral and brain sciences. (*A*) shows the distribution of the search terms among the articles in the sample. (*B*) shows the model organisms in each of the articles. Nonhuman rodent models included mice (n = 51), rats (n = 35), both rats and mice (n = 1), prairie voles (n = 3), Siberian hamsters (n = 2), degus (n = 1), gerbils (n = 1), and red squirrels (n = 1). The remaining five articles were on birds (collared flycatchers, n = 1; blue tits, n = 1), Barbary macaques (n = 1), pigs (n = 1), and *Drosophila* (n = 1).

Every article in our sample featured claims that males and females responded differently to a treatment, exposure, intervention, or other nonsex factor (e.g., genotype, age, season, presence of a health condition, etc.). To create our corpus, we searched the Web of Science Core Collection (Clarivate Analytics) for articles with terms such as “sex-specific” or “gender-dependent” in the title (Fig. 1*A*), restricting the results to articles published between 2019 and 2023 in fields related to the brain and behavior. Using a weighted sampling algorithm (26), we selected 200 articles from the resulting 1,341 (*SI Appendix*, Table S1 and *Supplemental Methods* and Dataset S1). The most common search term to appear in the titles was “sex-specific,” followed by “sex-dependent” and “gender-specific” (Fig. 1*A*). Half of the articles (n = 100) featured studies on human participants (Fig. 1*B*); of these, four also involved mice. Among the 100 articles on only nonhuman models, most (n = 95) were on rodents.

### Analytical Approaches.

**Fig. 2.**
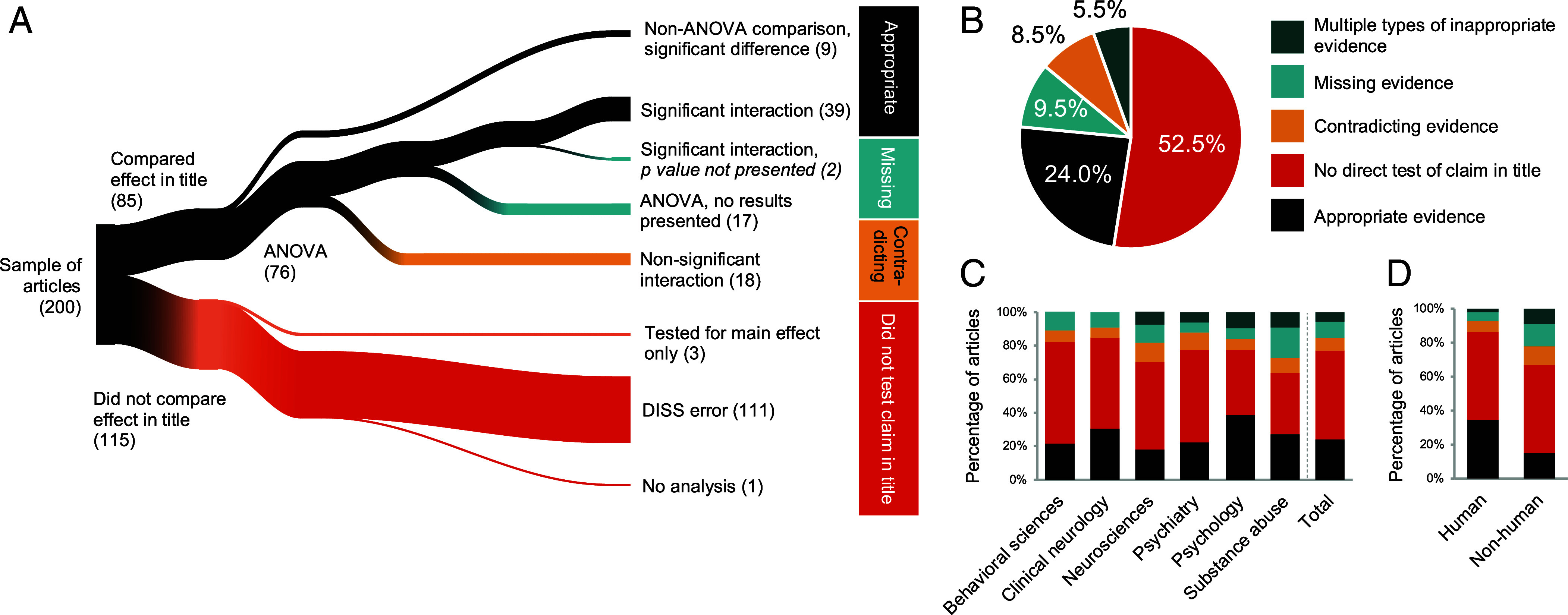
Analytical approaches supporting title claims of sex-dependent effects in the behavioral and brain sciences. The river plot (*A*) depicts the numbers of articles with each type of evidence. The approaches and colors shown on the right side of the panel correspond to those in the other panels (see Table 1 for categories and descriptions). In (*B*), articles in which authors used multiple types of inappropriate evidence (n = 11) are shown in dark teal (DISS and missing ANOVA results, n = 6; DISS and contradicting ANOVA results, n = 4; missing ANOVA results and contradicting ANOVA results, n = 1). The 11 articles with multiple types of inappropriate evidence are included in the river plot (*A*) as DISS (n = 10) or contradicting (n = 1). (*C*) Across the six focal research areas, articles in the field of psychology were the most likely to contain appropriate evidence of the claim of sex-dependent effects in the title, and those in neuroscience the least likely, compared separately with all other areas combined. Articles that were tagged with two research areas, for example, neurosciences and behavioral sciences, are included in both relevant columns. (*D*) Studies with only human participants were significantly more likely to include appropriate evidence than those with only nonhuman animals (*P* = 0.002). DISS: Difference In Sex-specific Significance; see Fig. 3.

We classified analytical approaches using criteria described in the Methods and Table 1; distributions are shown in Fig. 2. Of the 200 articles, 48 (24%) supported the sex-related title claim with appropriate evidence, meaning that the effect mentioned in the title was statistically compared across sex and the result of that comparison supported the claim (Table 1). In most of these articles (n = 39), a multifactorial ANOVA was performed with sex as a factor and a statistically significant interaction was reported between sex and the other factor(s). Seven articles reported a statistically significant difference between within-sex associations (e.g., within-sex Pearson correlations were compared with each other), and two compared other effect estimates, such as odds ratios, across sex. All these articles were coded as “appropriate.”

**Table 1.** Codes used for categorizing articles according to analytical approach

General analytical approach	Presentation of evidence	Evidence designation for graphs
1. Claim in title was tested statistically (e.g., in a multifactorial ANOVA with sex as a factor, comparison of associations, etc.)	1.1. Authors reported a *P* value indicating a significant interaction (a), significantly different within-sex correlation (b), or other appropriate statistical evidence (c) supporting the claim in the title	Appropriate evidence
	1.2. Authors reported a significant interaction with sex but did not report the relevant statistic (e.g., *P* value) 1.3. Authors claimed to have performed an appropriate test but did not report the result or state statistical significance, either significant or nonsignificant	Missing evidence
	1.4. Authors reported a *P* value indicating a nonsignificant interaction 1.5. Authors reported that the interaction was not significant but did not report the *P* value	Contradicting evidence
2. Claim in title was not tested statistically	2.1. Authors performed within-sex tests only (DISS error) 2.2. Authors tested whether sex explained variation in the overall model (e.g., main effect), then performed within-sex analyses 2.3. Authors performed no quantitative comparisons	No direct test of claim in title

Note: During the coding process, we also coded whether authors 1) conducted post hoc tests regardless of whether the interaction with sex category was significant or not significant, and/or 2) conducted sex comparisons within treated groups.

In the remaining 152 articles (76%), evidence used to support the title claim did not meet criteria to be considered appropriate. In these articles, a statistical sex comparison was claimed to have been conducted but the results were missing, the results were present but contradicted the claim, or the sex comparison was missing entirely. All such articles were coded as “inappropriate.” Thirty-seven of these took a multifactorial ANOVA approach; of these, two claimed a statistically significant interaction between sex and the other factor(s) but no *P* value was reported for that interaction. Seventeen articles claimed to have performed a multifactorial ANOVA with sex as a factor but failed to report both the *P* value and the result of the interaction test (significant or not). Together, these 19 articles missing the *P* value, the result, or both (8.5% of the sample of 200 articles) were coded as “missing” (Fig. 2). In 18 articles (9%), the interaction was explicitly reported as not statistically significant, indicating that the claim in the title was not supported by the data and that the null hypothesis—that the effect in question did not depend on sex—should not have been rejected. These articles, 15 of which included the nonsignificant *P* values for the interaction, were coded as “contradicting” (Fig. 2).

In the 76 articles using ANOVA, whether classified as appropriate or inappropriate, we noted whether post hoc tests of the effect were conducted within sex. Post hoc tests are typically predicated on first detecting a significant interaction, which provides evidence that the effect differs by sex (27). In 22 of the 76 articles, it appeared that all ANOVAs were followed by post hoc within-sex tests regardless of whether the interaction with sex was statistically significant, suggesting that the interaction test from the ANOVA had been disregarded. Because it is the interaction test, not the within-sex post hoc tests, that can indicate support for a sex-dependent effect, such post hoc tests did not affect our coding as appropriate or inappropriate; two of these articles were coded as “appropriate” because the title claim aligned with interactions that were significant. The rest were coded as “inappropriate” for other reasons, such as missing results of the ANOVAs.

**Fig. 3.**
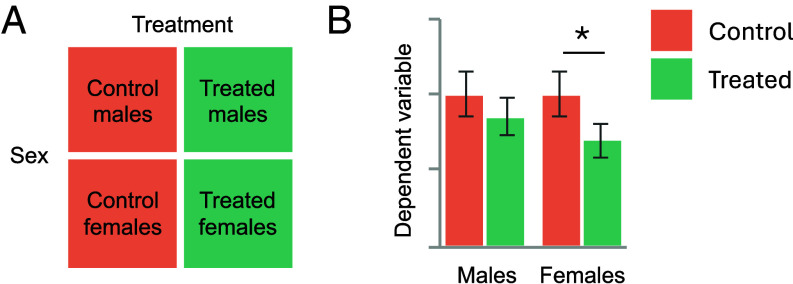
The “Difference in Sex-specific Significance (DISS) error” is an inappropriate analytical approach in which within-sex tests are conducted to determine whether males and females responded differently to a treatment (28). In a design such as (*A*) above, the DISS approach does not compare the sexes directly; instead, *P* values are generated within-sex and, if they are discrepant with respect to statistical significance (*B*), the effect is declared to be sex-dependent. But the DISS approach does not test whether the effect depends on sex; the sexes have not been compared statistically. To test for a difference in the treatment effect, we could test for a sex-by-treatment interaction; a significant interaction would provide evidence that the effect differed between females and males. For more information about the error and sample datasets, see Rich-Edwards and Maney (27).

In most articles (57.5%), no statistical test capable of supporting the title claim was conducted. These were coded as “no direct test.” In 111 of these 115 articles (55.5% of the 200 articles in the sample), the effect in the title was tested separately in females and males, and discrepant *P* values were interpreted as a sex difference. Inferring a sex-dependent effect from within-sex tests has been called the “difference in sex-specific significance” (DISS) error [(28); Fig. 3]. Because this approach does not directly compare the effect statistically across sex, it cannot provide appropriate evidence for a sex-dependent effect (9, 27–38). Similarly, in two of the 115 articles with no direct test, the sexes were compared within treated groups, disregarding the control groups. This error, which is similar to DISS, has been reported to occur in sex differences research (13, 36). In three of the 115 articles with no direct test of the claim in the title, sex was included in an omnibus test prior to within-sex analysis, but that model did not test for sex dependence of the effect in the title. Instead, the model tested for a sex difference in the outcome measure regardless of the other factors (e.g., main effect of sex), meaning that a significant sex difference did not relate to the claim in the title. The last of the 115 articles contained no quantitative analyses.

To assess whether inappropriate approaches were more common when the analyses were labeled as exploratory, we searched each article for the term “exploratory.” We found that 16 studies (8%) used the term to describe either the entire study or results related to the claim of sex dependence. Of these, 11 (68.8%) were coded as appropriate because sex by treatment interactions were tested, were significant, and the *P* values reported. The remaining five were coded as “no direct comparison–DISS” because the sexes were not statistically compared. These papers comprised only 4.3% of articles that were coded as “no direct comparison in the overall sample.”

### Research Area.

We next investigated the analytical approaches used within each of our focal research areas (Fig. 2*C*) as defined by the Web of Science tags (*SI Appendix, Supplemental Methods*). In our sample, psychology had the highest percentage of articles with appropriate statistical evidence (12 out of 31 articles, or 38.7%). The neurosciences had the lowest percentage of articles with appropriate statistical evidence at only 18.2% (14 out of 77 articles). In an exploratory analysis, we investigated the probabilities of using appropriate evidence in each research area individually, compared with all other areas; for example, we compared the set of articles tagged with neuroscience with those not tagged with neuroscience, and so on for each research area. In a model that included all six research areas, the set tagged with psychology was statistically significantly more likely to contain appropriate evidence for the claim in the title than articles outside psychology (OR = 4.705, 95% CI: [1.255, 18.127], *P* = 0.022). We found no other significant differences when comparing each area against all other areas (see *SI Appendix*, Tables S2 and S3 for complete results). We did not conduct pairwise comparisons between the areas because some articles were tagged with two research areas (*SI Appendix, Supplemental Methods*). We therefore do not draw conclusions about whether any research area was more or less likely to contain appropriate evidence than another specific area.

The DISS approach to a title claim was common in all six areas (*SI Appendix*, Fig. S1*A*). In our sample, psychiatry articles were most likely to feature a DISS approach to the title claim, at 61.2% (30 out of 49 articles) and substance abuse the least, at 40.9% (9 out of 22 articles). As we did above for appropriateness, we compared the prevalence of the error between the set of articles with a particular research area tag and those without it (*SI Appendix*, Fig. S1*A*). These tests showed that articles in psychology (OR = 0.271, 95% CI: [0.082, 0.869], *P* = 0.030) and substance abuse (OR = 0.227, 95% CI: [0.060, 0.824], *P* = 0.026) were less likely to employ a DISS approach to support the title claim than articles outside these areas (see *SI Appendix*, Tables S4 and S5 for complete results). Again, we did not conduct pairwise comparisons between research areas, so we do not claim that the DISS error is significantly more or less prevalent in any one area than in another.

### Human vs. Nonhuman Models.

Next, we investigated whether the appropriateness of analytical approaches was related to the choice of model organism (Fig. 2*D*). Of the 96 articles that included only human participants, 33 (34.3%) provided appropriate statistical evidence to support the claim in the title, whereas only 15 of 100 articles (15%) on nonhuman animals did so (nonhuman vs. human OR = 0.337, 95% CI: [0.165, 0.663], *P* = 0.002; Fig. 2*D* and see *SI Appendix*, Tables S6 and S7 for complete results).

The DISS error was the most common analytical approach both within the set of articles on human participants and in those on nonhuman animals. Of the 96 articles that included only human participants, 49 (51%) took a DISS approach to the title claim, as did 59 of the 100 articles (59%) on nonhuman animals. The odds of taking a DISS approach were 1.380 times higher for articles on nonhuman animals than for articles on human participants (95% CI: [0.786, 2.435]), but this difference was not statistically significant (*P* = 0.263) (*SI Appendix*, Fig. S1*B* and see *SI Appendix*, Tables S8 and S9 for complete results).

### Prestige and Impact.

**Fig. 4.**
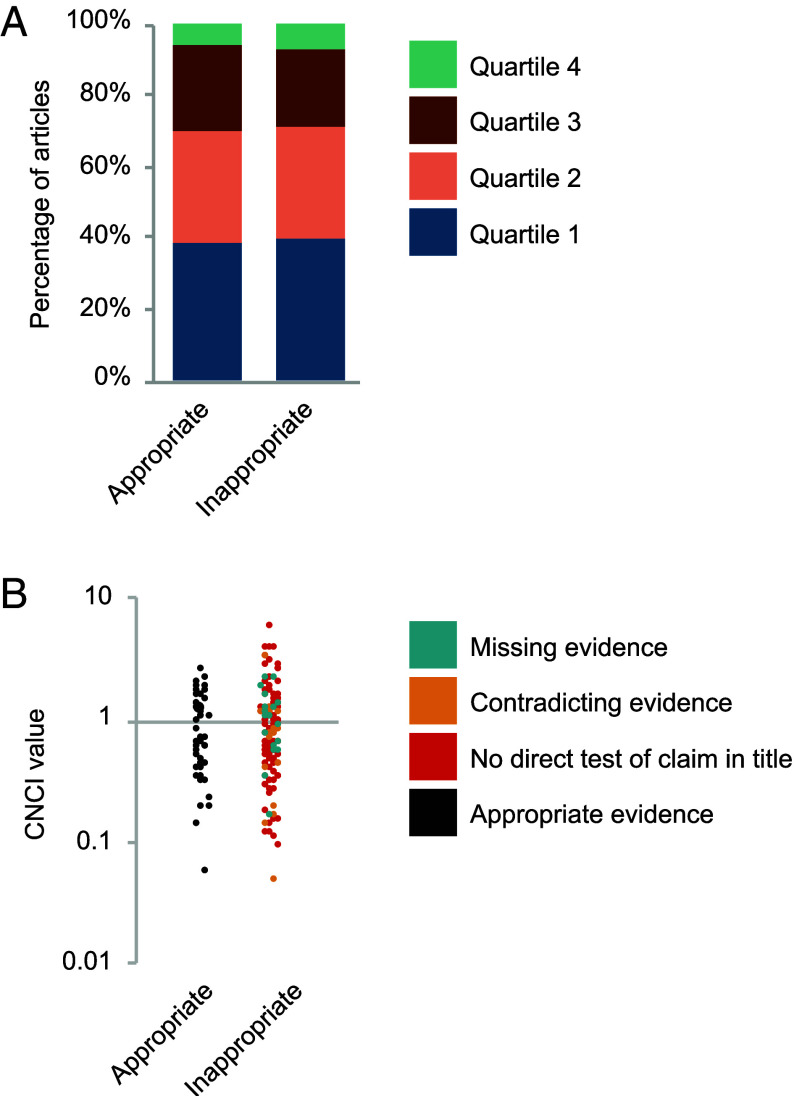
Associations between journal quartile or article impact and choice of analytic approach. The appropriateness of the analytic approach was associated with neither (*A*) journal quartile ranking from Journal Citation Reports (*P* = 0.942) nor (*B*) Category Normalized Citation Impact (CNCI) score from InCites (*P* = 0.859). Quartile ranking indicates the relative Journal Impact Factor within a research area, with Q1 being the highest. CNCI values greater than 1.0 indicate above-average citation impact, whereas values less than 1.0 indicate below-average citation impact.

We tested whether the choice of an appropriate analytical approach was associated with the prestige of the journal in which the article was published or the impact of the article itself. We operationalized prestige as the journal’s quartile from Journal Citation Reports, which is based on the impact factor of the journal (*SI Appendix, Supplemental Methods*). We found no association between the appropriateness of an article’s analytical approach and the quartile of the journal that published it (Fig. 4*A*; Reference group: Appropriate; OR = 1.028, 95% CI: [0.486, 2.177], *P* = 0.942). We also found no association between the use of the DISS approach and the quartile (Reference group: no DISS; OR = 0.836, 95% CI: [0.435, 1.607], *P* = 0.590; *SI Appendix*, Fig. S2*A*; see *SI Appendix*, Tables S10 and S11 for complete results). These findings indicate no compelling evidence that prestigious journals are significantly more or less discerning when it comes to rigor in the analysis of sex-based data than their less prestigious counterparts.

We operationalized the impact of the article using the Category Normalized Citation Impact (CNCI) score, an article-level citation metric provided by InCites (Clarivate Analytics). This metric represents the ratio of an article’s observed citation count to the expected citation count for publications of the same document type, publication year, and Web of Science subject category (39). We did not find an association between the appropriateness of the analytical approach and the article’s CNCI (Fig. 4*B*, 2.6% increase in inappropriate compared with appropriate, 95% CI: [−25.8, 30.9]%, *P* = 0.859). There was also no association between the use of the DISS approach specifically and the article’s CNCI (*SI Appendix*, Fig. S2*B*; 2.9% increase in DISS compared with no DISS, 95% CI: [−27.3, 21.5]%, *P* = 0.816; see *SI Appendix*, Tables S12 and S13 for complete results). These findings suggest that articles with appropriate evidence for claims of sex dependence are not significantly more or less impactful than articles without appropriate evidence.

### Claims of Sex-Dependent Effects over the Past Two Decades.

**Fig. 5.**
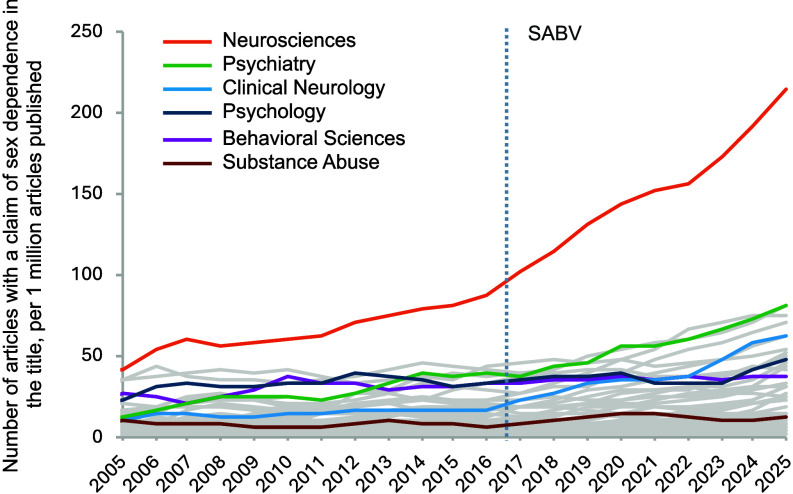
Title claims of sex-dependent findings over time. Each line represents articles with “sex-specific” or a similar term (Fig. 1*A*) in the title. The light gray lines represent 55 nonfocal research areas in Web of Science that do not pertain specifically to the brain or behavior (e.g., radiology, allergy, surgery; see Dataset S2 for a complete list). Each line shows the number of articles each year, normalized to the total number of articles published in all 61 research areas to control for a general increase in publication rates in all areas over time. We applied a 3-y centered moving average to reduce visual noise. All searches were conducted in January 2026, so the data for 2025 may underestimate publication counts. Source data are in Dataset S2.

To assess the extent to which sex-dependent effects are being emphasized in different research areas, we plotted the number of articles in our six focal areas that contained, in their titles, one of our search terms (e.g., “sex-specific”) each year from 2005–2025 (Fig. 5). For comparison, we did the same for 55 other Web of Science research areas related to health but not explicitly focused on the brain, cognition, or behavior (see Dataset S2 for the complete list of research areas). The annual number of articles with title claims of sex-dependent effects in neuroscience, normalized to the total number of articles published in all 61 research areas each year, was generally higher than in the other areas and increased by more than fivefold over the 20-y period. In 2025, there were 2.5 times as many articles with title claims of sex dependence published under the neurosciences tag than in the next-highest area, psychiatry.

We also calculated the probabilities that an article with a claim of sex dependence in the title would be tagged with each of the 61 research areas. Consistently across the entire 20-y period, an article with such a title claim was more likely to be a neuroscience article than any other area. By the time SABV was implemented in 2016, an article with a claim of a sex-dependent effect in the title was already twice as likely to be a neuroscience article than an article from the next most common area. By 2025, the chance had increased to three times as likely.

## Discussion

We report here that when articles in the behavioral and brain sciences include claims of sex-dependent effects in their titles, fewer than one-quarter (24%) present appropriate statistical support for the claim. In the majority of articles (56%), males and females were not compared statistically at all. Instead, independent tests were performed within each sex (DISS error, Fig. 3). Our data reveal a gap in scientific rigor in the face of mandates that were implemented explicitly to improve it.

On average, claims of sex-dependent effects are increasing in frequency over time (Fig. 5). Claims in the neurosciences are far outpacing other fields, which is a potentially important finding given that in our analysis of approaches, fewer than one in five claims of sex dependence in the neurosciences is supported by appropriate quantitative analyses (Fig. 2*C*). Because relying on within-sex tests causes strong bias toward false positive “findings” of difference (32, 40, 41), the high rate of inappropriate approaches in neuroscience could be at least partially responsible for the high number of positive findings being reported.

Appropriate approaches were more common in psychology than other areas, and more common in studies of human participants than in studies using nonhuman animals. These patterns likely reflect both disciplinary differences in statistical training and methodological differences between human and nonhuman research. In psychology, null hypothesis significance testing (NHST) has long been treated as a framework for statistical inference, with explicit attention to matching statistical tests to the claims being made and to the interpretation of comparisons among effects (42–44). In neuroscience, by contrast, several analyses have documented widespread inferential errors under NHST that arise not from the absence of statistical testing, but from failures to test precisely the claims being made (35), faulty interpretations of underpowered studies (45), and overreliance on dichotomous interpretations of *P* values (46). Studies on nonhuman models typically have lower power than human studies (45), increasing the risk that within-group analyses will fail to detect a true effect in one of the groups (32, 40, 41) and lead to a false conclusion of a sex-dependent effect.

High rates of inappropriate statistical approaches to sex-based data have been reported beyond the behavioral and brain sciences. Across many fields, within-sex tests are typically prioritized over tests for sex dependence (11, 47). Two small studies, focused broadly on the biological and biomedical sciences, found rates of DISS of around 70% among studies with factorial designs (13, 14). In an evaluation of meta-analyses of randomized control trials, Kastrati et al. (15) found that only about one in five claims of sex-dependent effects were accompanied by formal statistical tests of the effect. Kammula et al. (47) found that oncology clinical trials between 1972–2022 only rarely included a statistical comparison of effects between men and women; they attributed this “striking and discouraging” result to the tendency for clinical trials to report separate analyses by sex, which was by far the most common approach.

The reasons for choosing an inappropriate analytical approach are undoubtedly complex, but likely include misunderstandings of inference (19, 31, 32, 34, 35) or statistical power (36). Although underpowered studies are particularly vulnerable to DISS errors (32, 40, 41), some researchers believe the DISS approach *retains* power; one article in our sample stated that “Data were analyzed separately for each sex to retain adequate statistical power to identify the within-sex effects.”^*^ Power to detect the main effect of interest is highest when the data are included in a *single* model that includes sex; this single model can also test whether the effect depended on sex (27, 33, 35, 36, 48).

Choices of analytical strategies are likely related also to incentive structures (49). Incentives to perform within-sex tests have increased substantially with the implementation of policies such as SABV and SBGA, and some of the training materials provided by NIH and CIHR specifically endorse DISS approaches (49). For example, CIHR’s training course (1) states that interaction terms are generally not preferred because they are less “intuitive” and more “difficult to calculate and interpret” than the results of separate analyses. “When analyses are presented separately by sex,” CIHR explained, “this provides the clearest picture of where exposures might differ for men and women.” Our sample consisted entirely of articles published after SABV and SGBA went into effect, so our data do not speak to whether the policies themselves have directly affected analytical choices. Regardless of a potential effect of the policies, because the rate of claims has increased since they were implemented (Fig. 5) the number of unsupported claims is likely increasing as well.

Funders are not the primary stewards of appropriate analytical approaches; such gatekeeping is more important at the publication stage (11), and we might expect rates of appropriate analyses to be higher in top journals (50). We found, however, that articles with inappropriate approaches were just as likely to appear in highly ranked journals (Fig. 4*A*) or to be cited (Fig. 4*B*) as those with appropriate evidence. Many journals have adopted the SAGER guidelines, which recommend that all data be analyzed and presented disaggregated by sex (2, 51). Thus, as is the case for the SABV and SGBA policies, there is tension between compliance with the guidelines and maintaining rigor with respect to hypothesis testing, analytical approaches, and data interpretation.

### Limitations.

A significant sex-by-treatment interaction is only one of several forms of evidence that, when taken together, support the credibility of a sex-dependent effect. Other factors to consider include whether the effect is being compared across or within studies, whether the effect holds up across studies, and whether the effect and its direction were hypothesized a priori (52, 53). The absence of an a priori hypothesis can call into question even a statistically significant interaction (52). In a sample of acute care trials with high rates of prespecified sex-based analyses (91.3%), 82.6% tested for sex-by-intervention interactions (50), suggesting that the prespecification may increase the probability of a well-thought-out approach. In our study, we did not systematically track a priori hypotheses; only two of the 200 studies in our sample contained evidence of preregistration, such as a clinical trial number; both articles conducted comparisons between the sexes rather than using a DISS approach. In at least two articles, the authors stated explicitly that they had no a priori hypotheses about sex. Sixteen papers used the word “exploratory” to refer to the sex-based analysis. Yet, in each of these cases, the finding shifted from exploratory to title-worthy, illustrating how a sex difference can divert a study from its original purpose (27).

Although our study calls into question a substantial fraction of claims of sex-dependent effects, it does not overturn them. Only 8.5% of articles in our sample contained statistical evidence that clearly contradicted the claim in the title. The rest of the articles categorized as “inappropriate” either did not compare the sexes at all or did not report the results of the comparison. Therefore, we can claim only that the conclusions drawn in these articles are premature, not necessarily inaccurate. In a recent investigation of meta-analyses of clinical trials, Kastrati et al. (15) recalculated *P* values for sex-by-treatment interactions regardless of the analytical approach taken by the authors. Of 66 claims, only 27 (41%) had statistical support at *P* < 0.05. Reanalysis of the data in each article in our sample will be necessary to estimate how many claims hold up when the sexes are compared statistically.

The most frequent of our search terms, “sex-specific” (Fig. 1*A*), is sometimes used in confusing or incorrect ways. For example, we found that the term “sex-specific difference” nearly always referred simply to a sex difference, e.g., a difference in averages between females and males. Articles with that usage in the title were excluded. But we cannot rule out the possibility that some of the included articles mentioning a sex-specific effect, increase, association, etc. were in fact intending to refer to something else, such as a sex-specific part of the study. Such cases, the prevalence of which could depend on discipline, would have been rare in our sample; most articles using the term included clear interpretations of sex dependence, for example recommending sex-specific approaches to clinical practice. When referring to an effect that depends on sex, the term “sex-dependent” is more accurate than “sex-specific” or “males but not females;” the latter terms imply that the null hypothesis has been accepted in one sex.

### Recommendations.

For authors interested in providing strong support for claims of sex dependence, we recommend the following steps (27): First, regardless of statistical power, the effect should be compared statistically across sex. Relying instead on a DISS approach presents a high risk of false positive findings of “difference” (32, 40, 41) but can also cause large sex differences to be missed—for example, when effects go in opposite directions in males vs. females yet are not statistically significant within either sex (54). Typical tests include multifactorial ANOVA with sex as a factor, regression testing sex as a moderator, or a comparison of two associations (34). Note that a main effect of sex provides no evidence that the treatment effect depended on sex. Second, if the interaction between sex and treatment is statistically significant, that alone is sufficient evidence for a sex-dependent effect. Post hoc tests can provide information about the treatment effect within each sex, such as its direction, but the two *P* values from those tests cannot, alone, provide information about whether the effect depends on sex. Even when within-sex tests were planned a priori, those tests cannot provide evidence of a sex-dependent effect if the sexes were not directly compared statistically. Third, if the interaction is not statistically significant, then the null hypothesis of no sex difference cannot be rejected. A potential lack of power could be acknowledged. In all cases, if there was no a priori hypothesis about sex, findings of sex-dependent effects should be treated as exploratory (27, 52).

The above recommendations are most relevant when sex is treated as a binary categorical variable, as in all the articles in our sample. Opportunities to study sex-related variation in ways that move beyond simple categorical comparisons are increasing (55–59). These include modeling sex-related traits as continuous or multivariate characteristics or using probabilistic or exploratory frameworks that integrate multiple biological measures. Such approaches can capture variation that is obscured by strict binary categorization while reducing the tendency to overinterpret categorical differences.

Efforts to uncover meaningful sex differences, without analyses that produce meaningful evidence, risk producing a “literature of contradiction” (60) that muddles further progress and reduces the value of sex-inclusive research policies. In our sample of articles, unsubstantiated calls for sex-specific approaches to clinical practice were widespread. For example, authors called for sex-specific approaches to suicide prevention, stress-related psychiatric disorders, substance use disorder, and psychopathy, all without statistical comparisons across sex. In biomedicine, effects truly “specific” to one sex are likely rare (12, 25). Recommending different treatments for men and women, without convincing evidence of large sex differences, could create unnecessary or even discriminatory barriers to care, preventing patients from receiving the most beneficial interventions (21, 61, 62). Sex-inclusive biomedical research will fulfill its promise only when inclusion is matched by analytical rigor.

## Methods

All Methods are described in detail in the *SI Appendix*. We based our codes on those developed by Garcia-Sifuentes and Maney (13) to categorize analytical approaches to sex-disaggregated data (Table 1). For each article, we determined whether the effect of interest was statistically compared between females and males (e.g., via an interaction term or equivalent contrast). Articles meeting this criterion were classified as Approach 1 (compared); those that did not were classified as Approach 2 (not compared). Only articles reporting a statistically significant between-sex comparison of the effect (including a *P* value below alpha, 0.05 unless otherwise specified) were considered to provide appropriate support for the title claim. Articles that used both appropriate and inappropriate approaches to support the claim in the title were classified as inappropriate.

Within Approach 2, we further distinguished analyses limited to within-sex tests (difference in sex-specific significance, DISS), those including a main effect of sex without interaction, and those lacking quantitative comparisons. Two authors independently assessed all articles (~70% agreement); discrepancies were resolved by discussion, with additional adjudication by other authors as needed.

We evaluated associations between analytical approach and research area, model organism, journal quartile, and citation impact using regression models (logistic, ordinal, and linear as appropriate).

## Supplementary Material

Appendix 01 (PDF)

Dataset S01 (XLSX)

Dataset S02 (XLSX)

## Data Availability

All data supporting the findings of this study are provided in Datasets S1 and S2. The code used for data analysis is available at https://osf.io/59djk (63).

## Footnotes

^*^Our intention is to provide examples of misunderstandings and unsupported conclusions, not to attribute such to authors. Therefore, some examples are not formally cited.
