# Supplementary material for: Claims of sex-dependent effects in the behavioral and brain sciences are infrequently supported by valid statistical comparisons
Source: Proc Natl Acad Sci U S A. 2026 Aug 4;123(32):e2608703123. doi: 10.1073/pnas.2608703123 (PMC13462607; doi:10.1073/pnas.2608703123)
Supplement: Supplementary file 1 [file pnas.2608703123.sapp.pdf]

Supplemental Materials
Code and Supplemental data files available at: https://osf.io/59djk

Claims of sex-dependent effects in the behavioral and brain sciences are infrequently supported by valid statistical comparisons

Olivier, M. T.,[1] Brown, A. W.,[2,3] Chung, S.,[2,3] Vorland, C. J.,[4] and Maney, D. L.[1*]

[1]Department of Psychology, Emory University, Atlanta, GA 30322
[2]Department of Biostatistics, University of Arkansas for Medical Sciences, Little Rock, AR 72205
[3]Arkansas Children's Research Institute, Little Rock, AR 72202
[4]Department of Epidemiology and Biostatistics, Indiana University School of Public Health-Bloomington, Bloomington, IN 47403

*Correspondence to Donna L. Maney, dmaney@emory.edu

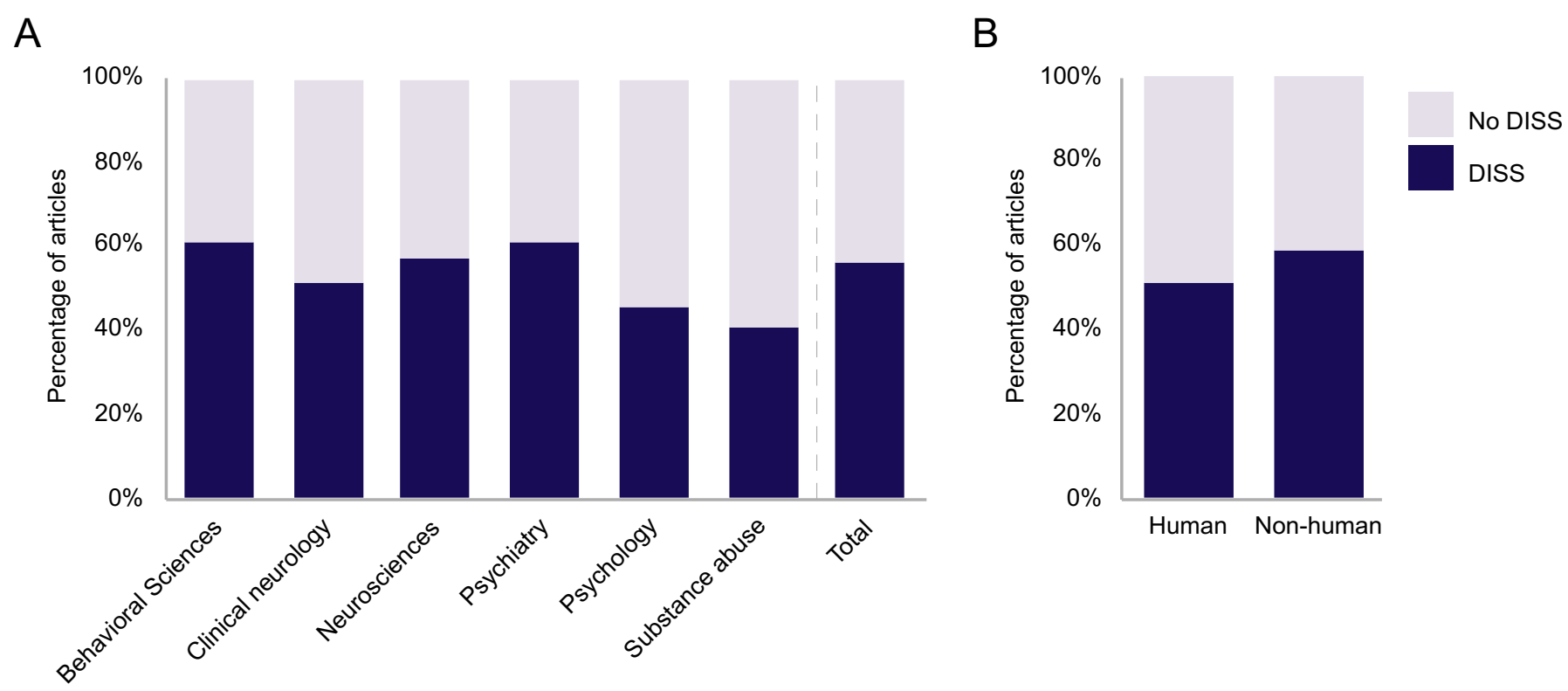

Fig. S1. Prevalence of DISS approaches across research areas and model organisms. (a) Across the InCites research areas, articles in the field of psychology and substance abuse were the least likely to feature DISS-based evidence of the claim of sex-dependent effects in the title. Analytic approaches in the "no DISS" category included appropriate as well as inappropriate, non-DISS approaches (see Fig. 2b). Psychology articles were significantly less likely to support the title claim using a DISS approach than non-psychology articles ($p = 0.030$); the same was the case for the area of substance abuse ($p = 0.026$). In the graph, articles that were tagged with two research areas are included in both relevant columns. (b) The prevalence of the DISS error did not differ between studies with only human participants and those with only non-human animals ($p = 0.263$).

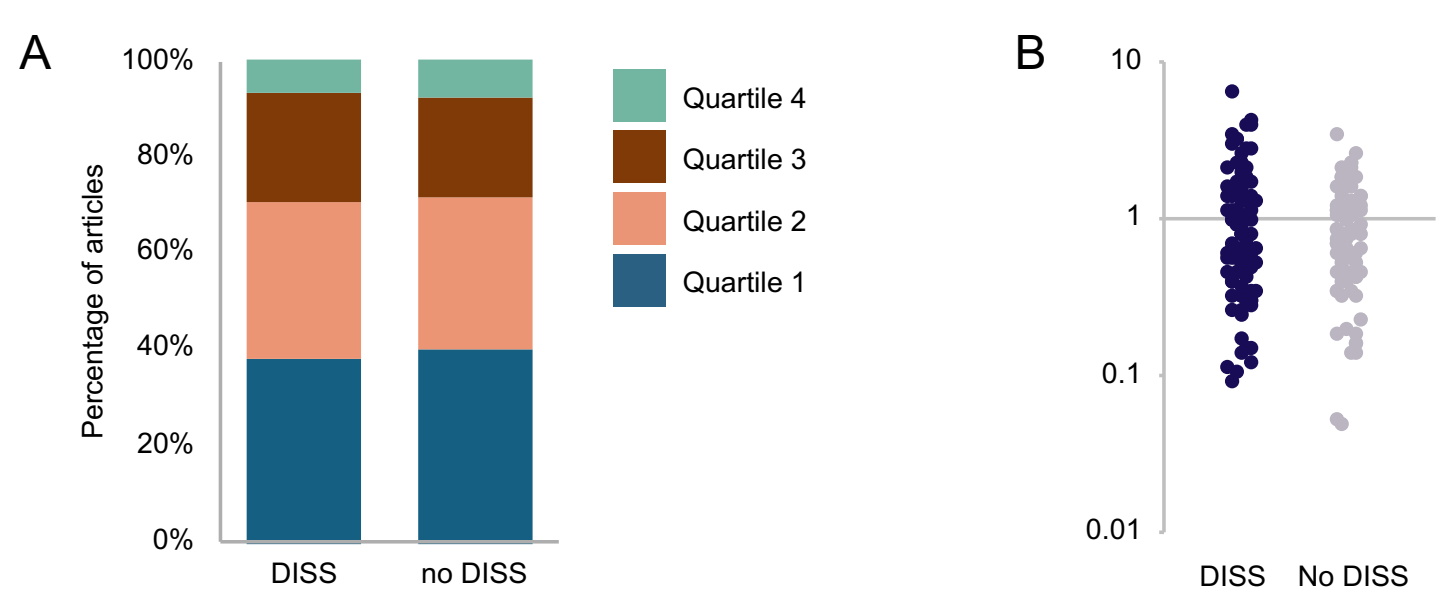

Fig. S2. Associations between the DISS approach and journal/article impact. We found no associations between the DISS approach and either (a) the journal's quartile ranking ($p = 0.590$) or (b) the article's Category Normalized Citation Impact (CNCI) score ($p = 0.816$). See the caption of Fig. 3 for explanations of these variables.

Table S1. Distribution of articles in the corpus across InCites research areas. Numbers in parentheses indicate the number of articles included in the final sample.

Research areas	*2019*	*2020*	*2021*	*2022*	*2023*	*TOTAL*
Behavioral sciences	12 (1)	9 (1)	14 (2)	12 (4)	14 (2)	61 (10)
Behavioral sciences; Clinical neurology; Psychiatry	0	0	1 (0)	0	0	1 (0)
Behavioral sciences; Neurosciences	18 (3)	19 (1)	28 (3)	20 (4)	20 (1)	105 (12)
Behavioral sciences; Neurosciences; Psychology	1 (0)	1 (0)	3 (0)	1 (0)	0	6 (0)
Behavioral sciences; Psychology	7 (1)	3 (1)	7 (2)	5 (1)	6 (1)	28 (6)
Clinical neurology	17 (6)	15 (2)	13 (3)	13 (5)	26 (11)	84 (27)
Clinical neurology; Neurosciences	9 (1)	20 (1)	15 (0)	17 (1)	23 (1)	84 (4)
Clinical neurology; Neurosciences; Psychiatry	1 (0)	4 (0)	5 (0)	3 (0)	1 (0)	14 (0)
Clinical neurology; Psychiatry	4 (0)	2 (0)	5 (0)	4 (1)	6 (1)	21 (2)
Clinical neurology; Neurosciences; Psychiatry; Psychology	0	0	0	0	1 (0)	1 (0)
Neurosciences	72 (7)	90 (8)	97 (11)	121 (8)	108 (12)	488 (46)
Neurosciences; Psychiatry	24 (2)	24 (2)	26 (2)	27 (3)	32 (6)	133 (15)
Neurosciences; Psychology	2 (0)	2 (0)	5 (0)	1 (0)	4 (0)	14 (0)
Psychiatry	19 (4)	12 (3)	20 (8)	28 (10)	19 (6)	98 (31)
Psychiatry; Psychology	4 (0)	6 (0)	5 (0)	2 (0)	12 (0)	29 (0)
Psychiatry; Psychology; Substance abuse	1 (0)	2 (0)	1 (0)	2 (0)	0	6 (0)
Psychiatry; Substance abuse	2 (0)	0	4 (1)	5 (0)	0	11 (1)
Psychology	24 (4)	22 (8)	20 (3)	14 (8)	23 (2)	103 (25)
Psychology; Substance abuse	3 (0)	3 (0)	0	0	1 (0)	7 (0)
Substance abuse	6 (3)	8 (5)	15 (4)	10 (4)	8 (5)	47 (21)
TOTAL	226 (32)	242 (32)	284 (39)	285 (49)	304 (48)	1,341 (200)

Table S2. Estimated probabilities that an article within a research area would have appropriate evidence. Probabilities were obtained via marginal predictions from the multivariable logistic regression model.

Research Area	*Probability of Appropriate (95% CI)*
Behavioral Sciences	0.199 [0.088, 0.391]
Clinical Neurology	0.302 [0.170, 0.477]
Neurosciences	0.177 [0.106, 0.280]
Psychiatry	0.221 [0.126, 0.359]
Psychology	0.387 [0.234, 0.565]
Substance Abuse	0.272 [0.127, 0.489]

Abbreviations: CI = Confidence Interval

Table S3. Summary of the multivariable logistic regression model assessing the association between six research areas and the use of appropriate evidence. Each research area was coded as a binary indicator.

Research Area	*OR*	*95% CI*	*p-value*
Behavioral Sciences	1.215	0.373, 3.651	0.734
Clinical Neurology	3.109	0.860, 11.188	0.080
Neurosciences	1.346	0.467, 3.863	0.587
Psychiatry	1.876	0.616, 5.569	0.258
Psychology	4.705	1.255, 18.127	0.022
Substance Abuse	2.811	0.619, 12.394	0.173

Abbreviations: CI = Confidence Interval, OR = Odds Ratio

Table S4. Estimated probabilities that an article within a research area would employ a DISS approach. Probabilities were obtained via marginal predictions from the multivariable logistic regression model.

Research Area	*Probability of Appropriate (95% CI)*
Behavioral Sciences	0.613 [0.420, 0.776]
Clinical Neurology	0.515 [0.348, 0.678]
Neurosciences	0.572 [0.459, 0.678]
Psychiatry	0.615 [0.471, 0.742]
Psychology	0.452 [0.289, 0.626]
Substance Abuse	0.409 [0.228, 0.618]

Abbreviations: CI = Confidence Interval, DISS = Difference in Sex-specific Significance

Table S5. Summary of the multivariable logistic regression model assessing the association between six research areas and employing a DISS approach. Each research area was coded as a binary indicator.

Research Area	***OR***	***95% CI***	***p-value***
Behavioral Sciences	0.916	0.355, 2.388	0.855
Clinical Neurology	0.382	0.121, 1.162	0.093
Neurosciences	0.495	0.196, 1.210	0.127
Psychiatry	0.687	0.268, 1.740	0.429
Psychology	0.271	0.082, 0.869	0.030
Substance Abuse	0.227	0.060, 0.824	0.026

Abbreviations: CI = Confidence Interval, DISS = Difference in Sex-specific Significance, OR = Odds Ratio,

Table S6. Estimated probabilities of appropriate evidence for human and non-human animal studies, respectively. Probabilities were obtained via marginal predictions from the univariable logistic regression model.

Model Organism	***Probability of Appropriate (95% CI)***
Human	0.344 [0.256, 0.444]
Non-human	0.150 [0.092, 0.234]

Abbreviations: CI = Confidence Interval

Table S7. Summary of the univariable logistic regression model assessing the association between model organism and the use of appropriate evidence. Human studies were used as the reference group.

Characteristic	***OR***	***95% CI***	***p-value***
(Intercept)	0.524	0.340, 0.792	0.003
Model Organism [Non-human]	0.337	0.165, 0.663	0.002

Abbreviations: CI = Confidence Interval, OR = Odds Ratio

Table S8. Estimated probabilities of a DISS approach in human and non-human animal studies, respectively. Probabilities were obtained via marginal predictions from the univariable logistic regression model.

Model Organism	***Probability of Appropriate (95% CI)***
Human	0.510 [0.411, 0.609]
Non-human	0.590 [0.491, 0.682]

Abbreviations: CI = Confidence Interval, DISS = Difference in Sex-specific Significance

Table S9. Summary of the univariable logistic regression model assessing the association between model organism and the DISS approach. Human studies were used as the reference group.

Characteristic	***OR***	***95% CI***	***p-value***
(Intercept)	1.043	0.698, 1.559	0.838
Model Organism [Non-human]	1.380	0.786, 2.435	0.263

Abbreviations: CI = Confidence Interval, DISS = Difference in Sex-specific Significance, OR = Odds Ratio

Table S10. Summary of the cumulative link mixed model assessing the association between the use of appropriate evidence and journal quartile rankings. Journal index indicators were included as covariates for adjustment. The outcome was the journal-specific quartile ranking for each article within each research area, accounting for index, and a random intercept for article ID was specified to account for within-article correlation.

Characteristic	*OR*	*95% CI*	*p-value*
Statistical Evidence [inappropriate]	1.028	0.486, 2.177	0.942
Index			
ESCI	1.000	---	
SCIE	0.899	0.268, 1.740	0.903
SSCI	1.152	0.190, 6.973	0.877

Abbreviations: CI = Confidence Interval, ESCI = Emerging Sources Citation Index, OR = Odds Ratio, SCIE = Science Citation Index Expanded, SSCI = Social Sciences Citation

Table S11. Summary of the cumulative link mixed model assessing the association between the DISS approach and journal quartile rankings. Journal index indicators were included as covariates for adjustment. The outcome was the journal-specific quartile ranking for each article within each research area, accounting for index, and a random intercept for article ID was specified to account for within-article correlation.

Characteristic	*OR*	*95% CI*	*p-value*
Statistical Evidence [inappropriate]	0.836	0.435, 1.607	0.590
Index			
ESCI	1.000	---	
SCIE	0.890	0.159, 4.965	0.8894
SSCI	1.120	0.184, 6.834	0.902

Abbreviations: CI = Confidence Interval, DISS = Difference in Sex-specific Significance, ESCI = Emerging Sources Citation Index, OR = Odds Ratio, SCIE = Science Citation Index Expanded, SSCI = Social Sciences Citation

Table S12. Summary of the linear regression model assessing the association between the use of appropriate evidence and the log-transformed Category Normalized Citation Impact (CNCI). Entries with CNCI values of zero were omitted because the logarithmic transformation is undefined at zero.

Characteristic	*Sympercent*	*95% CI*	*p-value*
(Intercept)	-35.179	-59.841, -10.518	0.005
Statistical Evidence [inappropriate]	2.562	-25.769, 30.893	0.859

Abbreviations: CI = Confidence Interval

Table S13. Summary of the linear regression model assessing the association between the use of appropriate evidence and the log-transformed Category Normalized Citation Impact (CNCI). Entries with CNCI values of zero were omitted because the logarithmic transformation is undefined at zero.

Characteristic	*Sympercent*	*95% CI*	*p-value*
(Intercept)	-31.942	-48.286, -15.598	<0.001
DISS Error	-2.890	-25.296, 21.516	0.816

Abbreviations: CI = Confidence Interval, DISS = Difference in Sex-specific Significance

Supplemental Methods

Article selection

We searched the Web of Science Core Collection for publications with titles containing phrases explicitly asserting sex- or gender-contingency. The search was conducted on May 24, 2024 with the search string: TI=("sex-specific" OR "sex-dependent" OR "gender-specific" OR "gender-dependent" OR "males but not females" OR "females but not males" OR "male but not female" OR "female but not male" OR "men but not women" OR "women but not men"). These search terms were selected *a priori* to maximize specificity rather than exhaustive coverage. We restricted results to articles published between 2019 and 2023 and excluded meeting abstracts, proceedings papers, review articles, early access papers, book chapters, editorial materials, and news items. This process yielded 4,870 articles.

Next, we filtered the results to include only articles in the behavioral and brain sciences. In Web of Science, each academic journal is tagged with at least one research area, which is assigned to all the articles published in that journal. We selected the following Web of Science research areas to include in our analysis: behavioral sciences, clinical neurology, neuroimaging, neurosciences, psychiatry, psychology, applied psychology, biological psychology, clinical psychology, developmental psychology, educational psychology, experimental psychology, mathematical psychology, multidisciplinary psychology, psychoanalysis psychology, social psychology, and substance abuse. Several major journals that publish articles across disciplines, such as Nature, are tagged as "multidisciplinary sciences" or "medicine, general internal" on Web of Science. To include relevant articles from these journals, we exported all articles to InCites, a tool that maintains the research area tags from Web of Science except for articles in the two multidisciplinary areas, which it sorts into more specific Web of Science research areas such as "neurosciences." Outside the two multidisciplinary areas, the research areas for each article remain the same between Web of Science and InCites.

We narrowed down the pool of articles to include only articles in the research areas listed above, leaving 1,341 articles remaining. Because of the large number of different Web of Science research areas related to psychology, we grouped the articles in those areas (psychology, applied psychology, biological psychology, clinical psychology, developmental psychology, educational psychology, experimental psychology, mathematical psychology, multidisciplinary psychology, psychoanalysis psychology, and social psychology) into one collapsed research area, which we called "psychology" for the present study. The research area "neuroimaging," which was represented by only six articles in the corpus, was grouped with neurosciences. Thus, the final corpus comprised articles from six research areas (Table S1): behavioral sciences, clinical neurology, neurosciences, psychiatry, psychology, and substance abuse.

Because there was a large disparity between the research areas with the largest and smallest numbers of articles in the corpus, we used an algorithm (1) that calculates and considers the weight of each research area to extract a sample of 200 articles (Table S1). This weighted sampling technique ensured that the joint distribution of research areas was balanced compared with the overall corpus while preserving the relative order of research areas from most to least frequent. One hundred sixty articles were tagged with a single research area and 40 were tagged with multiple research areas (e.g., both "neurosciences" and "behavioral sciences"; Table S1). No articles in the sample of 200 were tagged with more than two research areas.

To retrieve PDFs of each article in the sample, we used EndNote v.21. For PDFs not downloaded through EndNote, we searched PubMed or Google Scholar manually and downloaded the PDFs through the Emory University or Indiana University Bloomington libraries. For articles not available through either library, we used the Indiana University Bloomington InterLibrary Loan program.

As a first step in our coding process, we assessed articles to confirm each met inclusion criteria. We excluded four articles for being in a language other than English; 23 articles for not including a claim of a sex- or gender-dependent effect in the title (i.e., an article contained a search term in the title that did not refer to a sex or gender difference in the effect of another variable) and five articles for being the wrong type (two reviews, two unpublished manuscripts, and one qualitative study). In total, we excluded 32 articles during the first round of coding. To maintain the same distribution of articles across the six research areas

(see Table S1), we replaced each excluded article with another article from the same research area. When an excluded article was tagged with more than one research area, we replaced it with one tagged with the same two research areas. To identify replacement articles, we used a random number generator to prioritize articles within each research area (or set of two research areas, as relevant) for selection as replacements. During a second round of exclusions assessing the 32 new replacement articles, we ultimately excluded four articles for not including a claim of a sex- or gender-dependent effect in the title. We repeated the replacement process to identify four more articles. The four articles selected in this second round of substitution were kept during coding, bringing the total number of articles coded to 200.

Coding of articles

We based our codes on those developed by Garcia-Sifuentes and Maney (2) to categorize analytical approaches to sex-disaggregated data. For each article, we noted the approach used to test the effect in the title and assigned the article to at least one of the codes listed in Table 1. We assigned articles containing a mix of appropriate (Code 1.1) and inappropriate approaches (Codes 1.2 – 2.3) to the relevant codes comprising inappropriate approaches. Therefore, only articles that took no inappropriate approaches to support the title claim were coded as appropriate.

To assign codes for each article, we first asked whether the effect of interest was compared statistically between females and males directly — that is, sex was incorporated into the statistical model in a way that allowed statistical comparison of the effect of interest between the sexes. We coded articles meeting this criterion as General Analytical Approach #1 in Table 1. When authors tested the claim in the title statistically, they typically did so by testing for an interaction between sex and the other factor of interest (e.g., treatment) using a multifactorial analysis of variance (ANOVA). We considered this type of ANOVA — whether two-way, three-way, or having more factors — as having tested for interactions with sex so long as sex was one of the factors in the model. Occasionally, authors statistically compared the effect between males and females by employing other tests of interaction or moderation or a statistical comparison of within-sex associations (e.g., statistically comparing two within-sex correlations). We coded all such approaches into General Analytical Approach #1. Conducting post-hoc within-sex tests of the sex-dependent effect was not required for an article to be coded as appropriate.

After establishing that the effect in question was statistically compared across sex, we then assigned articles to one of five sub-codes that depended on whether the results of the comparison were reported or not, and whether the reported interaction or other relevant effect was significant or not. We assigned an article to the first of these sub-codes (Table 1, Code 1.1) if the p value was reported and was below alpha (we considered alpha to be 0.05 unless indicated otherwise in the article). Only articles in code 1.1 were considered appropriate; other articles were designated “missing” or “contradicting” (see Table 1). For articles that described ANOVAs, we noted whether within-sex post-hoc tests were conducted regardless of the statistical significance of the interaction test. This code was not exclusive of the other codes and did not affect whether an article was coded as appropriate or not.

When the effect in the title was not statistically compared between females and males, we assigned the article first to General Approach #2 (see Table 1), then into one of three sub-codes: the analytical approach was limited to within-sex analyses with no relevant between-sex statistical comparisons that could have supported the claim in the title (Code 2.1; DISS error; Fig. 2); the analytical approach included a test for a main effect of sex (but not interactions) before within-sex analyses (Code 2.2); no quantitative comparisons were performed in the study (Code 2.3).

Two coders (MTO and DLM) independently assessed each article. These coders chose the same codes, without discussion, ~ 70% of the time. In the cases of discrepant initial codes or uncertainty on the part of either coder, the coders discussed the article until reaching agreement. For eight articles, the best code for the analytical approach remained unclear after this discussion; in these cases, AWB read the article and discussed it with DLM and CJV until consensus was reached.

Journal and article metrics

To assess the relationship between the appropriateness of analytical approaches and the potential impact of the article, we took note of several metrics. First, we used Journal Citation Reports (Clarivate Analytics), which provides the journal's quartile ranking (Q1, Q2, Q3, or Q4) based on Journal Impact Factor. Because journals are given a new quartile ranking each year, we used the quartile the journal held the year the sampled article was published. Some journals did not have a quartile ranking in their citation reports for the year we were interested in; in this case, we excluded the sampled article from our analysis of the quartiles.

Journal Impact Factors and the associated quartiles are calculated within a journal's Web of Science subject category and indices, including the Science Citation Index Expanded (SCIE) and the Social Sciences Citation Index (SSCI). A journal tagged with a single research area could have up to two quartile measures, for example, one for SSCI and the other for SCIE. Likewise, a journal tagged with two research areas could have up to four quartile measures. When journals were tagged with additional, non-focal research areas that were not relevant to our study (e.g., developmental biology or endocrinology & metabolism), the quartiles associated with these research areas were not considered in our analysis. Twenty-one articles had discrepant quartiles either because they were tagged with multiple focal research areas (see Table S1 and Dataset S1) or because the quartile in one or more relevant areas was calculated in different indices, SCIE or SSCI. To make the graphs in Fig. 4a and S2a, we used the quartile for neuroscience when applicable (n = 15), SCIE over SSCI (n = 5), and clinical psychology over developmental psychology (n = 1). In our analysis of the data (below), we accounted for all quartiles assigned to a given article.

Second, we took note of each article's Category Normalized Citation Impact (CNCI), obtained from InCites. CNCI is an article-level citation metric that represents the ratio of an article's observed citation count to the expected citation count for publications of the same document type, publication year, and Web of Science subject category (3). We obtained article-level CNCI values by exporting DOI-identified records from Web of Science Core Collection to InCites, then downloading document-level metrics. We used the CNCI value assigned by InCites to each sampled article on Jan. 7, 2026 (InCites dataset updated Dec. 10, 2025; included Web of Science content indexed through Nov. 11, 2025).

Shared authorship among papers in the sample

We inspected the author lists of all 200 articles to identify potential overlap. Authors with identical full names were confirmed to be the same person by examining affiliations. Out of 200 articles, 30 shared at least one author with at least one other article in the sample. Eight of these 30 articles appeared in two different pairs and one appeared in three pairs, leading to a total of 20 unique pairs. Based on the sample distribution of the four analytical approaches (appropriate, missing, contradictory, and no direct test) over the entire sample of 200 papers, the probability that a randomly selected pair of articles shared an approach was 40.2%. In the sample with shared authors, eight of 20 pairs (40.0%) shared an analytical approach, a proportion similar to that in the overall sample of articles. Thus, we have no evidence that sharing an author increased the chances of also sharing an analytical approach, and we therefore felt comfortable treating articles as independent observations.

Data analysis

To test whether the use of appropriate statistical evidence is more common in any area compared with research outside that area, we fit a multivariable logistic regression model including all six research areas with an intercept-only logistic regression model. Because research areas were not mutually exclusive and the same article could be tagged with two, the categorical variable was represented using six binary indicator variables corresponding to the six research areas. In the full model, the odds ratio for each research area represents the change in the odds of an article containing only appropriate statistical evidence when it is tagged with that research area compared with articles not tagged with that research area. The same procedure was followed to test whether the use of the DISS approach was associated with a given research area.

To evaluate the association between model organism (human vs. non-human) and the presence of appropriate statistical evidence to support the claims in the title, we fit a univariate logistic regression that included a single indicator for non-human studies. After excluding four articles that were labeled as both human and non-human, the categories of ‘non-human’ and ‘human’ were mutually exclusive. The estimated odds ratio in this model represents the relative odds of an article on non-humans containing appropriate statistical evidence compared with articles on humans. The same procedure was followed to evaluate the association between model organism and the DISS approach.

To examine associations with quartile, we fit a cumulative link mixed-effects model with each article’s quartile, which articles inherit from the publishing journal, as an ordinal response and the presence of appropriate statistical evidence or DISS error as primary predictors in separate models. Because some of the journals were assigned multiple quartiles from different research areas and indices (SSCI or SCIE), a random intercept for each article was included to account for the within-article correlation. Index was included as a fixed effect. To evaluate the association between the analytical approach and the article’s impact, we fit a linear regression with each article’s CNCI as the response and the presence of appropriate statistical evidence or DISS error as the primary predictor. Log transformation was performed for CNCI prior to the model fitting to improve the normality of the residuals given the right-skewness and strict-positive nature of this measurement. Model coefficients were reported in the format of sympercent (4).

Assessment of claims over time

We examined how the prevalence of title claims of sex-dependent effects has changed over time in the behavioral and brain sciences. We used Web of Science and InCites, as described above, to search for articles published between 2005 and 2025 that were tagged with at least one of our six focal research areas (see Terminology and Scope, main text) and included one of our search terms (Fig. 2a) in the title. To illustrate how the rate of sex-dependent claims in our focal research areas changed in relation to other disciplines over time, we repeated the above process for articles tagged with 55 research areas that are biomedical in nature but do not focus on the brain or behavior specifically (e.g., oncology, gerontology, hematology; see Table S3). Research areas that did not relate to medicine, such as political science, geology, and ecology, were not included in our analysis. Articles that were tagged with one or more of our focal research areas as well as one or more of the 55 non-focal research areas were included in the relevant focal area(s) but excluded from the total count in the non-focal area(s) to prevent articles from appearing in both the focal and non-focal counts in the graph. This process applied as well to articles tagged with “multidisciplinary sciences” and “medicine general internal,” both of which were among the non-focal research areas. All articles with these two multidisciplinary tags were exported to InCites and moved to the relevant focal area if tagged by InCites as belonging to a focal area. All searches were conducted on January 10, 2026.

1. S. Chung, C. J. Vorland, D. L. Maney, A. W. Brown, A multivariate Bernoulli-based sampling method for multi-label data with application to meta-research. *arXiv* arXiv:2512.08371 (2025).
2. Y. Garcia-Sifuentes, D. L. Maney, Reporting and misreporting of sex differences in the biological sciences. *eLife* 10, e70817 (2021).
3. Clarivate, Category Normalized Citation Impact (CNCI). In InCites Indicators Handbook. Available at https://incites.zendesk.com/hc/en-gb/articles/25087312115601-Category-Normalized-Citation-Impact-CNCI. Accessed December 10, 2025 (2025).
4. T. J. Cole, Sympercents: symmetric percentage differences on the 100 loge scale simplify the presentation of log transformed data. *Stat. Med.* 19, 3109–3125 (2000).
